# Effects of normal saline versus lactated Ringer’s solution on organ function and inflammatory responses to heatstroke in rats

**DOI:** 10.1186/s40560-024-00746-y

**Published:** 2024-10-08

**Authors:** Lan Chen, Chang Liu, Zhaocai Zhang, Yuping Zhang, Xiuqin Feng

**Affiliations:** 1 Nursing Department, The Second Affiliated Hospital of Zhejiang University School of Medicine, 88 Jiefang Road, Shangcheng District, Hangzhou, 310009 Zhejiang China; 2 Department of Critical Care Medicine, The Second Affiliated Hospital of Zhejiang University School of Medicine, Hangzhou, Zhejiang China

**Keywords:** Heat stroke, Resuscitation fluids, Normal saline, Lactated Ringer’s solution, Inflammatory cytokines, Organ dysfunction, Apoptosis

## Abstract

**Background:**

Heatstroke is a life-threatening condition characterized by severe hyperthermia and multiple organ dysfunction. Both normal saline (NS) and lactated Ringer’s solution (LR) are commonly used for cooling and volume resuscitation in heatstroke patients; however, their specific impacts on patient outcomes during heatstroke management are poorly understood. Given that the systemic inflammatory response and multiple-organ damage caused by heat toxicity are the main pathophysiological features of heatstroke, the aim of this study was to evaluate the effects of NS and LR on the production of inflammatory cytokines and the functional and structural integrity of renal and cardiac tissues in a rat model of heatstroke.

**Methods:**

Fifty-five male Sprague‒Dawley rats were randomly divided into four groups: cold NS or LR infusion postheatstroke (4 ℃, 4 ml/100 g, over 10 min) and NS or LR infusion without heatstroke induction (control groups). Vital signs, arterial blood gases, inflammatory cytokines, and renal and cardiac function indicators, such as serum creatinine and cTnI, were monitored after treatment. Tissue samples were analysed via HE staining, electron microscopy, and fluorescence staining for apoptosis markers, and protein lysates were used for Western blotting of pyroptosis-related proteins.

**Results:**

Compared with LR-treated heatstroke rats, NS-treated heatstroke rats presented lower mean arterial pressures, worsened metabolic acidosis, and higher levels of IL-6 and TNF-α in both the serum and tissue. These rats also presented increased serum creatinine, troponin, catecholamines, and NGAL and reduced renal clearance. Histological and ultrastructural analyses revealed more severe tissue damage in NS-treated rats, with increased apoptosis and increased expression of NLRP3/caspase-1/GSDMD signalling molecules. Similar differences were not observed between the control groups receiving either NS or LR infusion. One NS-treated heatstroke rat died within 24 h, whereas all the LR-treated and control rats survived.

**Conclusions:**

NS resuscitation in heat-exposed rats significantly promotes metabolic acidosis and the inflammatory response, leading to greater functional and structural organ damage than does LR. These findings underscore the necessity of selecting appropriate resuscitation fluids for heatstroke management to minimize organ damage and improve outcomes.

**Supplementary Information:**

The online version contains supplementary material available at 10.1186/s40560-024-00746-y.

## Introduction

Heatstroke is a critical medical emergency characterized by extreme hyperthermia, often leading to multiple organ dysfunction syndrome (MODS) [1]. Among the most common complications are central nervous system impairment, circulatory failure, and renal dysfunction, which significantly impact the prognosis of heatstroke patients [2, 3]. The pathophysiological mechanisms underlying heatstroke-induced organ dysfunction are multifaceted and involve direct cellular injury from heat, endothelial activation, excessive inflammation, and impaired tissue perfusion [2, 4]. Direct cellular damage from heat exposure and the subsequent systemic inflammatory response are believed to play crucial roles [2].

Effective heatstroke management prioritizes rapid cooling and supportive care, with volume resuscitation being a crucial component [5, 6]. The administration of cold crystalloid fluids not only aids in cooling but also helps to expand the blood volume and replenish electrolytes lost due to hyperthermia. Normal saline (NS) and lactated Ringer’s solution (LR) are crystalloids that are commonly recommended and utilized in clinical settings. However, studies have shown that NS infusion may lead to hyperchloremia, which can enhance tubuloglomerular feedback, induce renal vasoconstriction, reduce renal blood flow and cortical perfusion, and ultimately impair renal function [7–9]. The high chloride content in NS exacerbates these effects, increasing the risk of renal damage, particularly in patients with pre-existing metabolic acidosis or renal impairment [10–12]. A retrospective cohort study by our team also suggested a correlation between the volume of NS administered in emergencies and the incidence of acute kidney injury (AKI) in heatstroke patients [13].

While extensive evidence has associated NS with adverse renal outcomes, most research has focused on renal blood flow and function rather than structural impacts. Moreover, infusions of high-chloride solutions such as NS have been linked to systemic complications such as metabolic acidosis [10] and heightened inflammatory responses, including the overexpression of inflammatory cytokines [14], which may increase mortality risk [15]. In contrast, LR has been associated with more favourable outcomes concerning electrolyte balance, acid‒base status [16, 17], and reduced inflammation [18]. Given the crucial role of systemic inflammation in heatstroke-induced organ damage, it is essential to explore whether different resuscitation fluids differentially influence the inflammatory response, potentially affecting heatstroke prognosis.

To address these gaps, we investigated the inflammatory effects of NS and LR in a heatstroke model using Sprague‒Dawley rats. We assessed blood and tissue indicators and evaluated the functional and structural integrity of major organs (kidney and heart) via biochemical markers and pathological analysis. Our hypothesis posited that NS exacerbates the production of inflammatory cytokines and results in more severe renal and cardiac damage than does LR. In addition, to further elucidate the effects of the fluids under various pathophysiological conditions, we included normal rats receiving either NS or LR as controls. We also targeted the NLRP3/caspase-1/GSDMD pathway in renal tissue to explore the mechanisms underlying inflammatory regulation and cell death.

## Materials and methods

### Animal preparation and housing

**Fig. 1:**
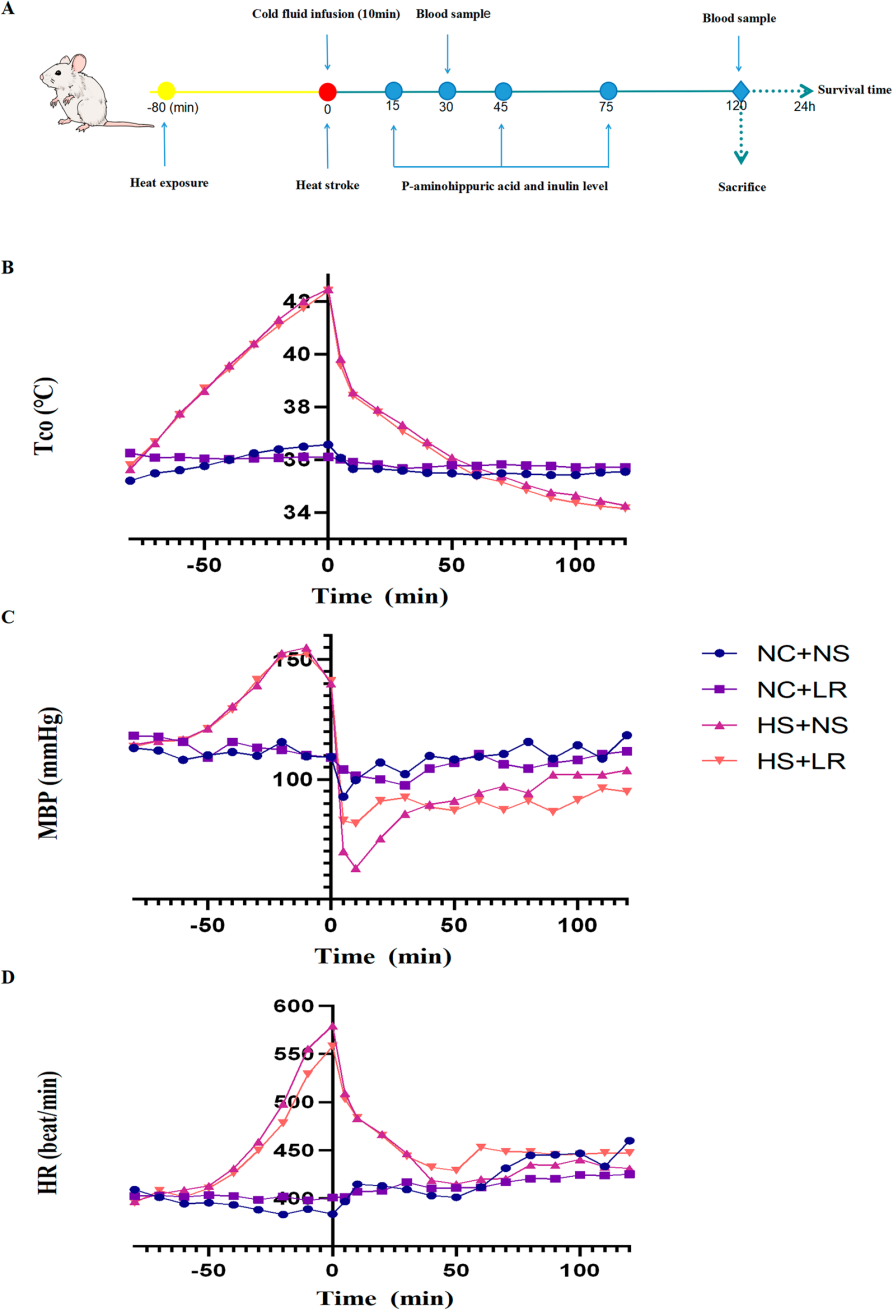
Experimental procedures and physiological variables. **A** Overview of the experimental process. **B**, **C**, **D** Trends in vital signs over time from heat exposure to 2 h post-heatstroke onset, including Tco, MAP, and HR. Following heatstroke onset, the MAP decreased sharply in both heatstroke rat groups, with a more pronounced and severe decrease observed in the HS+NS group. Significant differences in MAP were noted at 5, 10, and 20 min post-event (**C**). NC + NS, *n* = 13; NC+LR, HS+NS, and HS+LR, *n* = 14. *Tco* core body temperature, *MAP* mean arterial pressure, *HR* heart rate

The timeline of the study is presented in Fig. 1A. Adult male SD rats (350–400 g) were obtained from Zhejiang Weitong Lihua Experimental Animal Technology Co., Ltd. (Licence No: MA28BDKP-5). The rats were individually housed at 20–25 ℃ and 45–50% humidity under a 12-h light/dark cycle. The rats had free access to standard rodent feed and water for 1 week of acclimatization prior to the experiments.

### Ethical approval and euthanasia procedure

All experimental protocols, including animal care and handling, were approved by the Ethics and Welfare Committee of Animal Experimentation at the Second Affiliated Hospital, Zhejiang University School of Medicine (Approval ID: AIRB-2023-1677). This approval confirms adherence to the ethical standards and guidelines for animal research. Postexperiment, the surviving rats were euthanized via an overdose of sodium pentobarbital, which was administered to ensure painless death, which was consistent with the ethical guidelines for animal euthanasia.

### Surgical procedures and physiological monitoring

#### Anaesthesia and surgical preparation

Anaesthesia was induced in the rats via intraperitoneal injection of sodium pentobarbital (40 mg/kg), which was carefully monitored to maintain an appropriate depth of anaesthesia throughout the procedures. Sodium pentobarbital is known to affect cardiovascular parameters temporarily; hence, additional doses were administered judiciously, particularly to avoid critical periods 10 min before and 30 min after heat stroke onset, to prevent data interference.

#### Vascular access and monitoring

Following anaesthesia, a 22 G cannula was inserted into the right common carotid artery for continuous physiological monitoring and blood sampling. Another cannula was inserted into the tail vein for fluid administration. Physiological variables such as the mean arterial pressure (MAP) and heart rate (HR) were continuously recorded via a bioinformation acquisition system (Chengdu Taimeng, BL-420 N). Core body temperature (Tco) was recorded every 10 min via a veterinary anal thermometer.

### Heatstroke induction

The rats were placed on a heating pad set to 43 ℃ to simulate heatstroke conditions. Current studies use two criteria for heatstroke modeling in rats: a Tco exceeding 42 ℃ or 43 ℃, and a decrease in MAP. However, the standards for MAP reduction are inconsistent, varying from a decrease from the peak, a drop of 25 mmHg, or a reduction to below 30 mmHg [19–21]. In our research, we observed that after heat exposure, the MAP of rats gradually increased and fluctuated after reaching its peak, making the “start of decline” criterion challenging to apply. To ensure model consistency and minimize the impact of hypotension on organ damage, we defined heatstroke onset as an increase in Tco to 42 ℃, accompanied by a decrease in MAP of at least 10 mmHg, typically occurring within 75–80 min, as shown in Fig. 1B. After heatstroke induction, body weight was measured again, and weight loss due to heat exposure was calculated based on the difference from the baseline weight.

### Sample size calculation

This study is a randomized controlled trial in which rat inflammatory markers were used as the basis for sample size calculations. According to previous studies [22], when the effects of HCL and LR on inflammatory markers in septic rats were compared, 6–7 rats per group achieved good statistical power. As this study divided each group into two subgroups on the basis of blood collection time, the final sample size for each group was determined to be 12–14 rats. During the experiment, if carotid artery cannulation failed or a rat died before the completion of infusion, it was removed from the study. After removal, the number of rats in each group remained no less than 12, or if the data available for analysis in each group were more than six, no additional animals were supplemented.

### Experimental design and group allocation

The rats were randomized into the following four groups via a random number table method:

Normothermic control with NS (NC+NS, *n* = 13): These rats were maintained under normothermic conditions with NS infusion.

Normothermic control with LR (NC+LR, *n* = 14): These rats were maintained under normothermic conditions with LR infusion.

Heatstroke with NS resuscitation (HS+NS, *n* = 14): These rats were subjected to heatstroke and treated with NS for cooling and fluid resuscitation.

Heatstroke with LR resuscitation (HS+LR, *n* = 14): These rats were subjected to heatstroke and treated with LR for cooling and fluid resuscitation.

### Resuscitation protocols

The NC+NS and NC+LR groups were maintained at a steady Tco of 36–36.5 ℃ using a water circulation blanket for 80 min, followed by NS or LR infusion at room temperature. This protocol was designed to simulate the administration of fluid without the confounding effects of heat stress, serving as a control to assess the baseline impact of NS under normothermic conditions.

The HS+NS and HS+LR groups were exposed to 43 ℃ conditions until heatstroke onset. Immediately following heat stroke induction, the rats were removed from the heat source and placed in an ambient environment of 20–25 ℃. Resuscitation fluids at 4 ℃ (NS for the HS+NS group and LR for the HS+LR group) were administered.

### Fluid composition and administration

NS contained 0.9% saline with 154 mmol/L chloride (Shuanghe Pharmaceutical).

LR contains sodium chloride, sodium lactate, potassium chloride, and calcium chloride (chloride at 108 mmol/L, lactate at 27 mmol/L) (Weigao Pharmaceutical).

*Administration Protocol* Despite the existence of well-established methods for modelling heatstroke in rats, no literature has defined the standards for the volume and rate of cooling and volume resuscitation fluids in heatstroke rats. Excessive infusion volumes can result in death due to volume overload, whereas insufficient volumes do not allow for the observation of the pathophysiological effects of different fluid types on heatstroke rats.

We based the infusion volume calculation in this study on the chloride ion input. On the basis of the conversion rules of body surface area between humans and rats [23], following established recommendations of cooling and volume resuscitation in heatstroke patients [2, 5], and considering the blood volume of the rats, our research team conducted a thorough investigation to determine the appropriate infusion volume. We ultimately established the following infusion protocol for this study: 4 mL per 100 g body weight over 10 min using a constant rate infusion pump (KellyMed, ZNB-XD).

### Postinfusion monitoring and analysis

Blood samples for biochemical and inflammatory marker analyses were collected at 30 and 120 min postresuscitation. Blood inulin and p-aminohippuric acid concentrations were measured at 15, 45, and 75 min after infusion. Half of the rats underwent immediate euthanasia for organ and histological examination to assess renal and cardiac integrity and apoptosis. The levels of inflammasome-related proteins in kidney tissue were quantified via Western blot analysis. The remaining rats were observed for a 24-h period to assess survival.

### Adaptation of methodology for renal function assessment

Initially, our strategy aimed to assess renal blood flow (RBF) and the glomerular filtration rate (GFR) via traditional clearance methods for p-aminohippuric acid and inulin. However, owing to insufficient urine output from heat-stroked rats, which compromised the reliability of these measurements, we adapted our approach. We opted to estimate the RBF and GFR on the basis of the serum concentrations of these markers, providing a viable alternative to reflect changes in renal function more consistently.

For the revised approach, we administered infusion fluids containing inulin (1.0 g/L) and p-aminohippuric acid (0.2 g/L). Blood samples were collected at 15, 45, and 75 min postinfusion, from the onset of heat stroke, and stored at − 80 ℃ until analysis. The serum levels of these compounds were quantified via ELISA kits (Shanghai Fankew Industrial Co.) following the manufacturer’s guidelines.

#### Blood sample analysis for organ function and inflammatory markers

##### Collection and storage

Blood samples were drawn at 30 and 120 min post-heatstroke induction. Immediate analyses for arterial blood gases and electrolytes were conducted (EDAN, Shenzhen Libang Precision Instrument Co., Ltd.), whereas samples for prothrombin time (PT) and complete blood count (CBC) were analysed within 4 h of collection. The remaining blood samples were centrifuged at 3000 rpm for 15 min to separate the serum, which was then stored at − 80 ℃ until further analysis.

#### Biochemical analysis and inflammatory markers

Biochemical parameters, including PT, blood urea nitrogen (BUN), serum creatinine (Scr), and alanine aminotransferase (ALT), were measured via an automatic biochemical analyser (LWC400, Shenzhen Lanyun Medical Device Technology Co., Ltd.). The CBC was determined with a fully automatic blood analyser (Jiangxi Tekang Technology Co., Ltd.). Serum levels of IL-6, TNF-α, neutrophil gelatinase-associated lipocalin (NGAL), cardiac troponin I (cTnI), and catecholamines were quantified via specific ELISA kits (Wuhan Kelu Biotechnology Co., Ltd. and Shanghai Fankew Industrial Co.), following the manufacturers' protocols.

#### Tissue sample analysis for organ function and inflammation

The tissues were rinsed, weighed, and homogenized in PBS to ensure complete disruption. The homogenates were centrifuged, and the supernatants were used for further analysis of inflammatory and damage markers.

#### Histological and ultrastructural evaluation

##### Histology

Both kidney and heart tissues were fixed, embedded in paraffin, and sectioned into 4-μm slices for histological examination. These sections were stained with haematoxylin and eosin (H&E) and evaluated microscopically via a blinded semiquantitative scoring method to assess organ damage. Renal damage was quantified via a scale designed for acute renal failure [24], with a focus on 100 cortical tubules across at least 10 different areas per kidney. The scoring criteria included tubular epithelial flattening, cell membrane bleb formation, brush border loss, cytoplasmic vacuolation, cell necrosis, tubular lumen obstruction, and interstitial oedema, with a maximum possible score of 10 points per tubule. Cardiac tissue damage was assessed by examining cellular features such as eosinophilic cytoplasm and nucleus, cytoplasmic vacuolation, inflammatory cell infiltration, and interstitial vessel congestion and haemorrhage via a standardized scoring system [25].

#### Transmission electron microscopy (TEM)

Ultrastructural analysis of kidney tissues was performed via TEM after fixation with 2.5% glutaraldehyde and 1% OsO4. The tissues were subjected to a series of dehydration and embedding processes, sectioned, double-stained with uranium acetate and lead citrate, and examined under a HITACHI HT7650 microscope.

#### Evaluation of apoptosis in renal cortex tissue sections

Apoptosis was assessed via a one-step TUNEL assay kit (HKI0011, Haoke). The tissues were fixed, embedded, and sectioned. After being deparaffinized in xylene, the tissue sections were rehydrated through a graded ethanol series. Following deparaffinization and rehydration, the sections underwent antigen retrieval via an EDTA solution and were allowed to cool. A TUNEL reaction mixture was subsequently applied, and the sections were incubated at 37 ℃. After incubation, the sections were washed and stained with DAPI for nuclear visualization. Finally, the prepared sections were mounted and digitally scanned via a fluorescence microscope (Eclipse Ci-L, Nikon, Japan). Renal cortex tissue images were captured at 20 × magnification via SlideViewer microscope software. We performed TUNEL staining on kidney specimens from four rats in each group. Apoptotic cells, indicated by red fluorescence, were quantified in 10 randomly selected high-power fields and averaged by an observer blinded to the samples.

#### Western blot analysis of protein expression in kidney tissue

For Western blot analysis, kidney tissues were first rinsed, minced, and homogenized in RIPA buffer (C1053, ApplyGene) supplemented with phosphoprotease inhibitors. After centrifugation, the supernatant was collected for protein quantification via a BCA protein assay (P1511, ApplyGene). The proteins were then separated by SDS‒PAGE (P1200, Solarbio) and transferred onto PVDF membranes (ISEQ00010, Millipore) for immunoblotting. The membranes were incubated with primary antibodies against (1:500, Immunoway, YT5382), caspase-1 (1:500, Immunoway, YT5743), GSDMD (1:1000, Immunoway, YT7991), and β-actin (1:5000, Immunoway, YM3028), followed by visualization via a chemiluminescent substrate (P1010, ApplyGene). The developed films were digitally processed to quantify protein band intensities via ImageJ software.

### Statistical analysis

The data are presented as the means ± SDs. For quantitative variables, one-way analysis of variance (ANOVA) was employed, followed by the least significant difference (LSD) method for pairwise comparisons. A random effects model was applied to analyse repeated measurements. In this study, we compared the differences in various indicators between the two heatstroke groups (HS+NS vs. HS+LR) and between the two normal control groups (NC+NS vs. NC+LR). This was done to more clearly delineate the differences in the effects of NS and LR under different pathophysiological conditions. Statistical analyses were performed via Empower software and GraphPad Prism 9.0.0. A *P* value less than 0.05 was considered to indicate statistical significance.

## Results

### Overall experimental conditions

In the NC+NS group, one rat was excluded because of bilateral carotid artery cannulation failure, leaving 13 rats. The other three groups each contained 14 rats. In the HS+NS group, one blood sample for p-aminohippuric acid and inulin testing was accidentally spilled, reducing the sample size by one.

### Early stage organ dysfunction in heatstroke model rats

#### Physiological responses after heat stress

Following heat exposure, rats exhibit adaptive physiological responses, including dilation of skin vessels and contraction of visceral vessels, which promote heat dissipation. This response, however, precipitated notable hypotension, especially during the cooling and volume replacement phase after heatstroke onset [2, 21]. As shown in Fig. 1, there was a gradual increase in Tco with extended heat exposure, accompanied by increases in MAP and HR. Symptoms such as shortness of breath and moistness around the mouth were noted when Tco surpassed 39 ℃. MAP markedly decreased at Tcos above 42 ℃, with the most significant decrease recorded within the first 10 min after heatstroke—(81.62 ± 14.17 mmHg in the HS+LR group compared with 63.00 ± 15.00 mmHg in the HS+NS group). The MAP recovery was gradual thereafter. An average body weight loss of 3.5 g was noted after heat exposure.

#### Inflammatory response and biochemical markers

Heatstroke induces organ dysfunction primarily through thermal injury and inflammation. Early indications of acidosis, coupled with persistently elevated levels of cTnI, catecholamines, BUN, and Scr, reflect acute renal and cardiac dysfunction. The increase in ALT and NGAL levels further highlights the widespread impact on multiple organs (Tables 1, S1). In the HS+NS and HS+LR groups, more severe tissue damage and increased apoptosis were observed (Figs. 3, 4 and 5). In addition, the IL-6 and TNF-α levels were significantly elevated early after heatstroke (Table 1), and a pronounced increase in the expression of inflammasome-related proteins was detected (Fig. 6). One rat in the HS+NS group died 16 h postexposure, and survival was monitored for 24 h.

**Table 1:** Comparison of experimental characteristics between four groups

Variables	NC+NS (*n* = 13)^※^	NC+LR (*n* = 14)^※^	HS+NS (*n* = 14)^※^	HS+LR (*n* = 14)^※^	*P* value^§^	*P* value^#^
Electrolytes and ABG						
Chloride (mmol/L)						
0.5 h	105.29 ± 2.06	100.57 ± 1.52	115.57 ± 3.65	109.00 ± 3.37	0.004	< 0.001
2 h	102.50 ± 4.46	101.57 ± 2.07	109.71 ± 4.72	107.86 ± 2.41	**–**	**–**
PCO_2_ (mmHg)						
0.5 h	44.73 ± 5.20	46.04 ± 4.76	28.53 ± 4.41	34.99 ± 4.11	**–**	0.016
2 h	37.87 ± 3.00	41.94 ± 4.45	36.77 ± 7.55	37.49 ± 9.82	**–**	**–**
Base excess						
0.5 h	− 0.69 ± 1.20	− 0.20 ± 1.82	− 10.07 ± 2.09	− 4.01 ± 1.04	**–**	< 0.001
2 h	0.88 ± 1.01	1.17 ± 0.59	− 4.89 ± 3.22	− 2.40 ± 3.47	**–**	–
Lactate (mmol/L)						
0.5 h	0.70 ± 0.22	0.70 ± 0.25	2.34 ± 0.74	1.40 ± 0.30	**–**	0.001
2 h	0.68 ± 0.27	0.44 ± 0.08	0.53 ± 0.26	0.59 ± 0.23	**–**	**–**
HCO_3-_^−^ (mmol/L)						
0.5 h	24.36 ± 1.35	25.51 ± 1.67	15.47 ± 1.74	20.99 ± 1.32	**–**	0.000
2 h	25.10 ± 0.81	26.94 ± 1.46	20.46 ± 3.36	22.36 ± 3.80	**–**	**–**
Routine blood test						
WBC (10^9/L)						
0.5 h	8.78 ± 4.22	6.83 ± 2.40	13.55 ± 2.42	11.94 ± 1.96	**–**	**–**
2 h	10.56 ± 2.97	7.25 ± 2.08	12.69 ± 2.35	8.69 ± 2.56	0.025	0.006
Cardiac function						
cTnl (pg/mL)						
0.5 h	41.76 ± 12.13	55.49 ± 9.62	174.33 ± 24.03	114.78 ± 31.75	**–**	< 0.001
2 h	46.07 ± 11.18	59.36 ± 9.50	169.40 ± 63.60	106.11 ± 26.05	**–**	0.003
Catecholamines (ng/mL)						
0.5 h	167.36 ± 39.76	169.34 ± 21.30	398.39 ± 86.16	292.81 ± 81.71	**–**	0.005
2 h	165.02 ± 16.39	181.78 ± 30.74	329.74 ± 62.01	256.76 ± 30.10	**–**	0.002
cTnl (Cardiac tissue) (pg/mL)	172.73 ± 50.26	179.84 ± 43.46	582.58 ± 135.32	417.29 ± 140.71	**–**	0.006
Catecholamines (Cardiac tissue) (ng/mL)	305.52 ± 32.63	313.74 ± 29.35	737.37 ± 89.02	493.58 ± 32.35	**–**	< 0.001
Renal function						
Scr (umol/L)						
0.5 h	35.99 ± 1.62	36.19 ± 2.40	53.16 ± 7.42	43.93 ± 1.93	**–**	0.000
2 h	35.70 ± 2.44	38.24 ± 2.82	42.73 ± 2.38	42.76 ± 2.69	**–**	**–**
NGAL (ng/mL)						
0.5 h	1.46 ± 0.31	1.57 ± 0.32	2.32 ± 0.47	1.73 ± 0.56	**–**	0.016
2 h	1.39 ± 0.29	1.54 ± 0.25	3.60 ± 0.71	2.02 ± 0.23	**–**	< 0.001
NGAL (Renal tissue) (ng/mL)	5.78 ± 1.76	5.87 ± 0.64	10.31 ± 1.45	7.26 ± 0.74	**–**	< 0.001
Inflammation Indicators						
IL-6 (pg/mL)						
0.5 h	21.92 ± 3.91	20.42 ± 1.77	67.61 ± 20.37	45.90 ± 17.94	**–**	0.007
2 h	22.94 ± 4.15	24.49 ± 4.05	72.98 ± 27.68	36.11 ± 9.13	**–**	< 0.001
TNF-α (pg/mL)						
0.5 h	90.75 ± 16.46	93.49 ± 7.06	186.64 ± 26.76	134.73 ± 24.60	**–**	< 0.001
2 h	93.66 ± 11.81	90.71 ± 10.27	182.71 ± 49.08	129.99 ± 15.99	**–**	0.002
IL-6 (Cardiac tissue) (pg/mL)	144.78 ± 22.13	143.43 ± 20.81	371.72 ± 116.80	301.90 ± 82.37	**–**	**–**
TNF-α (Cardiac tissue) (pg/mL)	190.91 ± 18.11	196.87 ± 18.62	525.28 ± 98.10	350.95 ± 52.45	**–**	< 0.001

All specimens are blood samples unless otherwise noted. Catecholamines, cTnl, NGAL, IL-6, and TNF-α are measured using ELISA kits. Data are represented as the mean ± SD

Group Sample Sizes: NC+NS: n = 13 (7 in 0.5 h, 6 in 2 h); NC+LR, HS+NS, and HS+LR: n = 14 (7 in 0.5 h, 7 in 2 h); Tissue sample (all groups): *n* = 7

Comparisons: P^§^: NC+NS vs. NC + LR; P^#^: HS+NS vs. HS+LR

*ABG* arterial blood gases, *cTnI* cardiac troponin I, *IL-6* interleukin-6, *NGAL* neutrophil gelatinase-associated lipocalin, *TNF-α* tumor necrosis factor-alpha, *WBC* white blood cell count

The above results verified the effect of hyperthermia on the internal environment of the body, as well as the role of hyperthermia in activating inflammation and damaging the structure and function of organs.

### Effects of different fluid infusions on control rats and heatstroke model rats

#### Effects on control rats

Compared with LR, NS infusion increased blood chloride levels at 0.5 h postinfusion in the normal control group (105.29 ± 2.06 vs. 100.57 ± 1.52) and white blood cell (WBC) counts at 2 h postinfusion (10.56 ± 2.97 vs. 7.25 ± 2.08) (Table 1). However, no significant differences in other test parameters or pathological evaluations were observed between these two groups in this study. These results suggest that in normal rats, short-term NS infusion elevates the blood chloride concentration without significantly promoting inflammation or causing damage to organ function and structure.

#### Effects on heatstroke model rats

##### Effects on the internal environment and mortality

Following heatstroke, the MAP decreased sharply in both heatstroke rat groups, with the decrease being more pronounced and severe in the HS+NS group. Significant differences in MAP were observed at 5, 10, and 20 min postevent (all *P* < 0.05) (Fig. 1C). One rat in the HS+NS group died within 24 h after heatstroke. NS infusion led to more drastic changes in parameters reflecting the internal environment [higher serum chloride (115.57 ± 3.65 VS. 109.00 ± 3.37 mmol/L, *P* < 0.001) and signs of metabolic acidosis—lower base excess (BE) (− 10.07 ± 2.09 VS. − 4.01 ± 1.04, *P* < 0.001) and higher lactate (2.34 ± 0.74 VS. 1.40 ± 0.30 mmol/L, *P* = 0.001)—with lower PCO_2_ (28.53 ± 4.41 VS. 34.99 ± 4.11 mmHg, *P* = 0.016) and HCO3^−^ (15.47 ± 1.74 VS. 20.99 ± 1.32 mmol/L, *P* < 0.001)] (Table 1).

#### Impact on renal and cardiac function

**Fig. 2:**
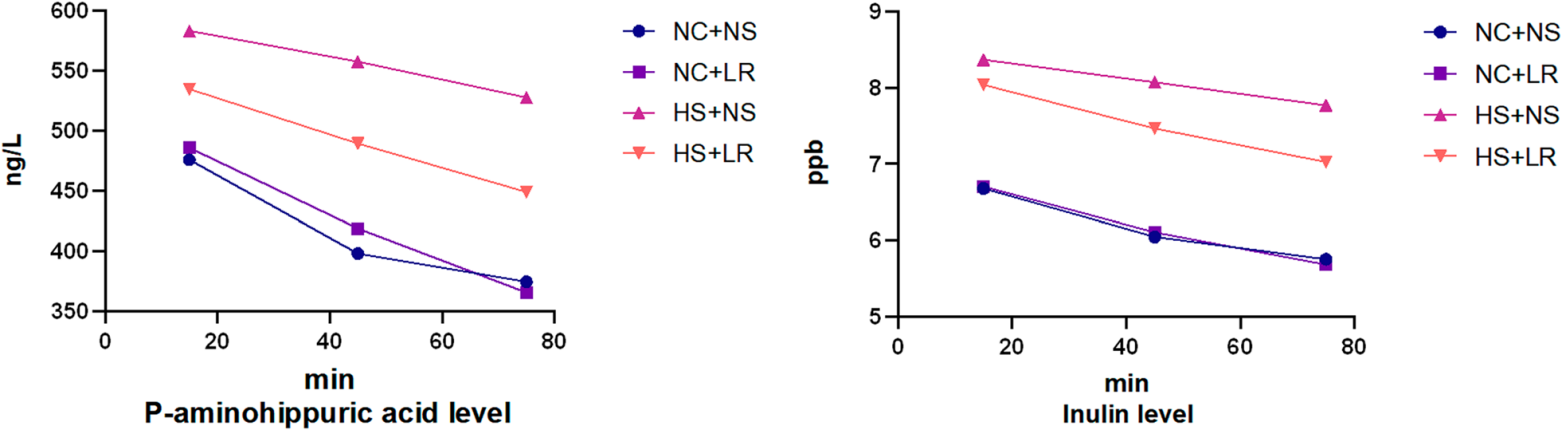
Serum concentrations of para-aminohippuric acid and inulin over time. Measured using ELISA kits; NC+NS and HS+NS, *n* = 13; NC+LR and HS+LR, *n* = 14. As time progressed, the concentrations of para-aminohippuric acid and inulin in the blood of all groups exhibited a decreasing trend. Compared to the HS+LR group, the HS+NS group showed lower concentrations at each time point, with the differences becoming more pronounced over time. Statistically significant differences between the two groups were observed for inulin at 45 and 75 min, and for para-aminohippuric acid at 75 min

The impact of different fluids on RBF and the GFR was assessed by evaluating the temporal changes in serum para-aminohippuric acid and inulin concentrations. NS administration was associated with higher serum concentrations of p-aminohippuric acid and inulin than LR at all measured time points (Fig. 2). The difference between the HS+NS and HS+LR groups increased over time, with statistically significant differences emerging at 45 and 75 min for inulin and at 75 min for p-aminohippuric acid (*P* < 0.05). In addition, elevated levels of Scr (53.16 ± 7.42 vs. 43.93 ± 1.93 µmol/L, *P* < 0.001) and NGAL [serum (2.32 ± 0.47 VS. 1.73 ± 0.56 ng/mL, *P* = 0.016) and tissue (10.31 ± 1.45 VS. 7.26 ± 0.74 ng/mL, *P* < 0.001)] were observed in the HS+NS group, indicating more pronounced renal injury in this group than in the HS+LR group (Table 1). These results indicate that, compared with LR, NS reduces the RBF and GFR and increases the risk of renal function impairment in heatstroke rats.

In addition, the HS+NS group presented elevated levels of cTnI and catecholamines both in the serum and tissue (all *P* < 0.05), indicating the exacerbation of cardiac dysfunction.

#### Effects on renal and cardiac tissue structures

##### Impact on renal tissue structure

**Fig. 3:**
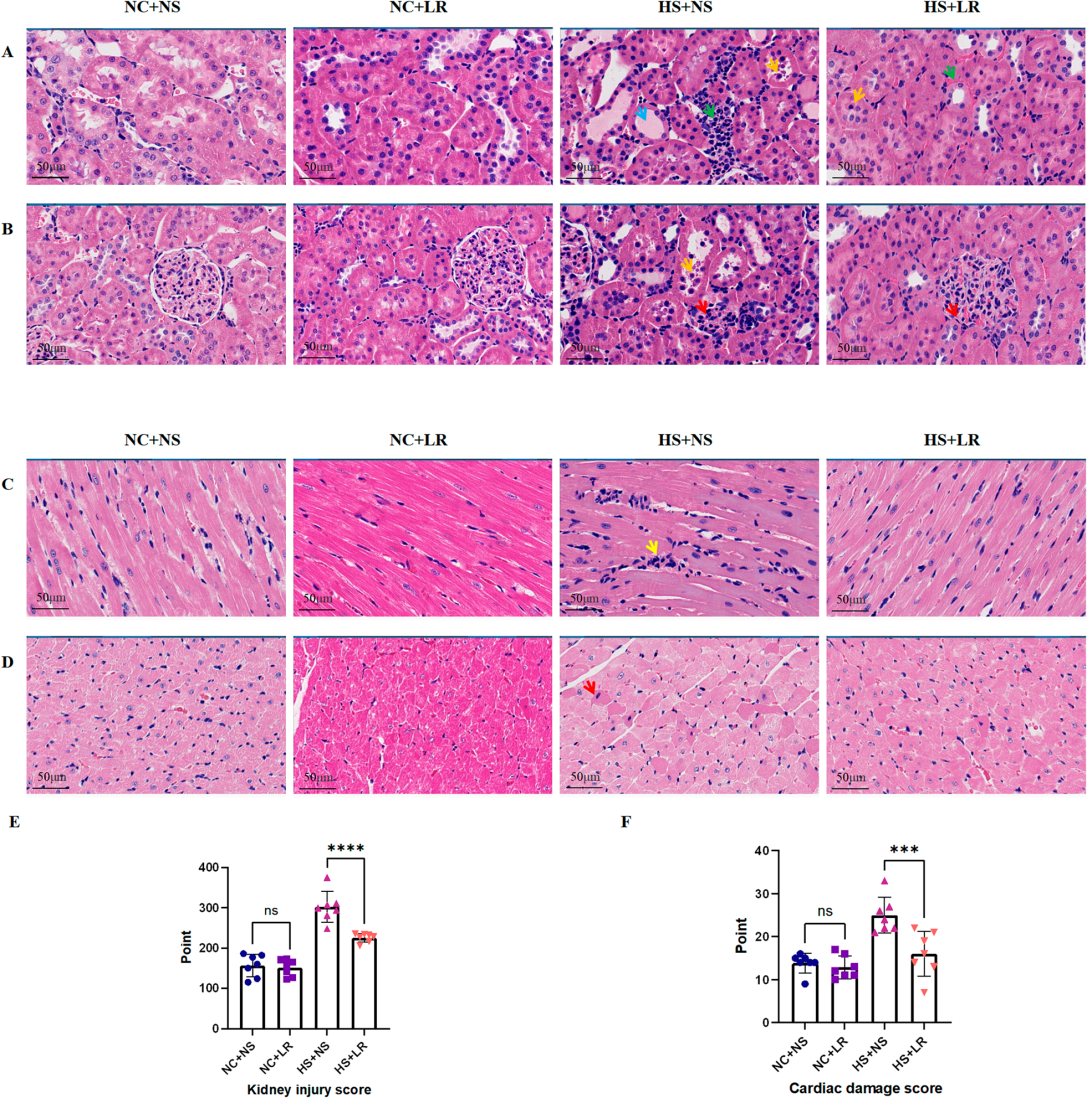
Histological evaluation. **A**, **B** Renal histopathology (H&E staining): HS+NS Group: Pale red, homogeneously stained casts are evident within renal tubular lumens (Blue arrow). Tubular epithelial cells appear flattened with signs of degeneration and necrosis (Yellow arrow), alongside interstitial inflammatory cell infiltration (Green arrow) (**A**). Irregularly shaped glomeruli and collapse of Bowman’s capsule are observed (Red arrow) (**B**). HS+LR Group: Tubular epithelial cell swelling and luminal occlusion are noted, with minor degeneration and necrosis (Yellow arrow), and interstitial congestion (Green arrow) (**A**). Enlarged glomeruli and occlusion of Bowman’s spaces are present (Red arrow) (**B**). NC+NS and NC+LR Groups: Tubular epithelial cells and glomeruli remain intact with only mild interstitial congestion. **C**, **D** Cardiac histopathology (H&E staining): HS+NS Group: Congestive inflammatory cell infiltration (Yellow arrow) (**C**), with some cardiomyocytes showing hyperstained nuclei and enhanced cytoplasmic eosinophilia (Red arrow) (**D**). The HS+LR group demonstrates less severe manifestations. **E** Comparative kidney injury score. **F** Comparative cardiac damage score. Scale bar = 50 µm. All groups *n* = 7. Data are represented as the mean ± SD. ****P ≤ 0.0001, ***P ≤ 0.001

HE staining and electron microscopy were used to evaluate the effects on organ structure. Histopathological examination revealed more extensive renal damage in the HS+NS group (Fig. 3A, B), with epithelial cell flattening, swelling causing lumen occlusion, and significant necrosis, including brush border degradation. There was pronounced inflammatory infiltration and glomerular damage, including ruptured glomeruli and renal capsule collapse. The HS+LR group displayed milder damage, such as epithelial swelling and mild interstitial congestion. The kidney injury scores in the HS+LR group were significantly lower than those in the HS+NS group (Fig. 3E), suggesting that LR had a protective effect on renal structures in heatstroke rats.

**Fig. 4:**
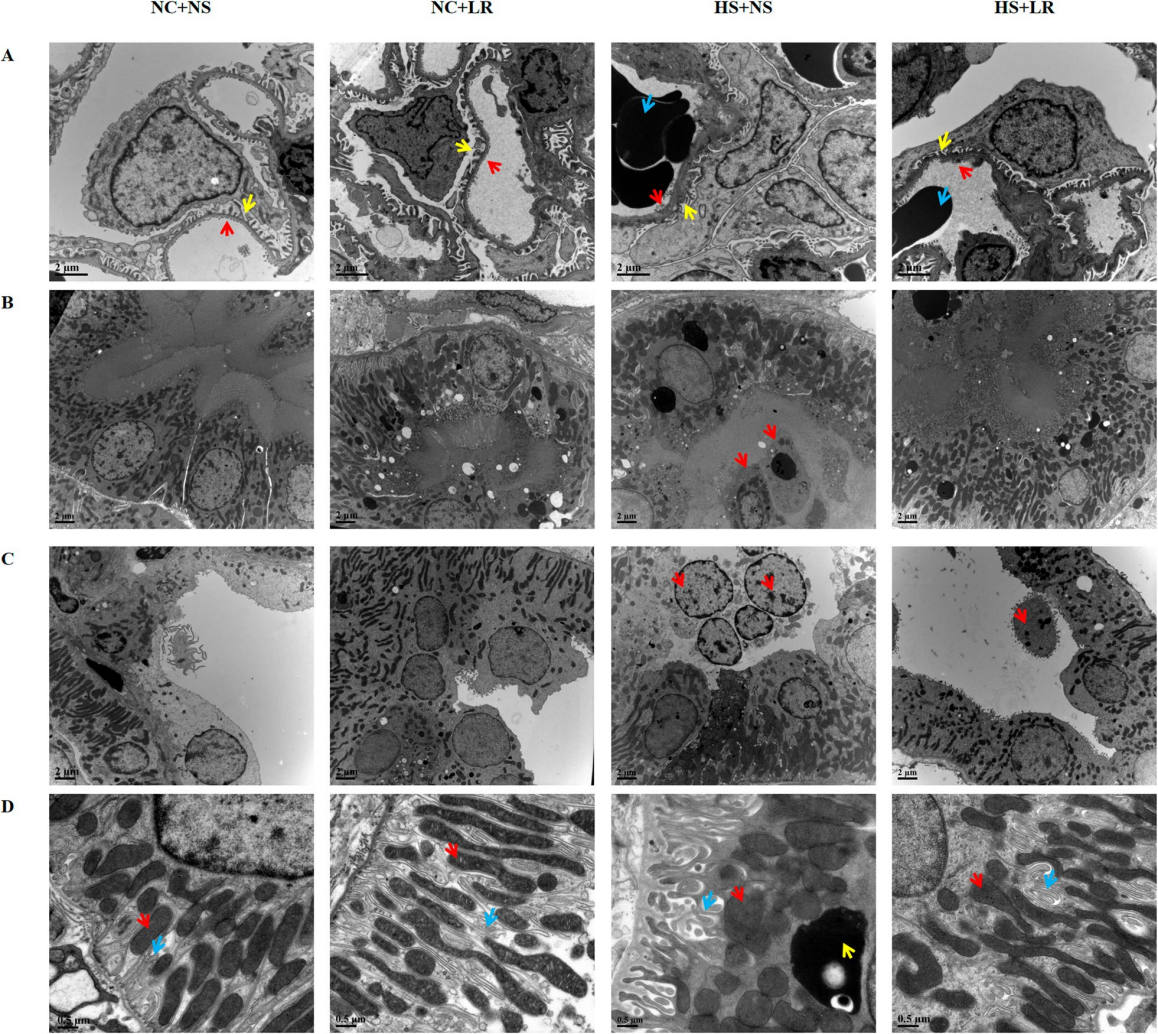
Renal electron microscopy images. **A** Glomerulus: In the NC+NS and NC+LR groups, the basement membrane of the glomeruli was uniformly thickened, with podocytes showing intact foot processes and only occasional mild fusion. The endothelial fenestrations were normal, and the capillary lumens were open. In the HS+NS group, podocyte foot processes were adherent, collapsed, and extensively fused, exhibiting microvillus-like changes. The endothelial cells were swollen, fenestrations were absent, and numerous red blood cells were retained within the capillary lumens. In the HS+LR group, podocyte fusion was partial, and endothelial fenestrations were absent but to a lesser extent than in the HS+NS group. Yellow arrow: Podocyte foot processes; Red arrow: Endothelial fenestrations; Blue arrow: Red blood cells. **B**, **C** Renal Tubule: In the NC+NS and NC+LR groups, the morphology of the renal tubules was normal, with occasional cellular debris within the lumens. In the HS+NS group, there was acute tubular injury, with lumens filled with necrotic cellular debris and visible nuclei. The HS+LR group exhibited less tubular damage than the HS+NS group, with fewer cellular debris in the lumens. **B** Proximal convoluted tubule; **C** Distal convoluted tubule; Red arrow: Necrotic cell nuclei and cellular debris. **D** Organelle: In the NC+NS and NC+LR groups, the structure of the renal tubular epithelial cells was normal, with well-developed basolateral infoldings and numerous mitochondria interspersed among them. In the HS+NS group, mitochondrial swelling was evident in the renal tubular epithelial cells, with uniform lightening of the matrix, shortened and reduced cristae, and a decrease or disappearance of matrix granules. There was an increased presence of lysosomes, with a reduction in other organelles such as the endoplasmic reticulum and Golgi apparatus. The infoldings in the basal membrane of epithelial cells were shortened. In the HS+LR group, the damage to renal tubular epithelial cells was less severe than in the HS+NS group. Red arrow: Mitochondria; Blue arrow: Basement membrane folds; Yellow arrow: Lysosomes

In the HS+NS group, ultrastructural examination revealed severe cellular damage: the glomerular endothelial cells were swollen, the capillaries were congested with red blood cells, and the podocyte foot processes exhibited extensive fusion and effacement (Fig. 4). The mitochondria within renal tubular epithelial cells were swollen with damaged cristae, and some exhibited membrane rupture, filling the tubular lumens with cellular debris. Conversely, the HS+LR group exhibited these pathological changes to a lesser extent, indicating that LR had a milder impact on the renal ultrastructure than NS did.

#### Impact on cardiac tissue structure

Cardiac tissues in the HS+NS group also presented more severe damage, including enhanced nuclear hyperstaining and increased cytoplasmic eosinophilia in cardiomyocytes, which is indicative of increased cell injury and inflammation (Fig. 3C, D). Conversely, cardiac tissues in the HS+LR group presented significantly less damage, as evidenced by lower histological scores (Fig. 3F), suggesting that LR had a protective effect on cardiac structures in heatstroke rats.

#### Cell apoptosis in renal tissue

**Fig. 5:**
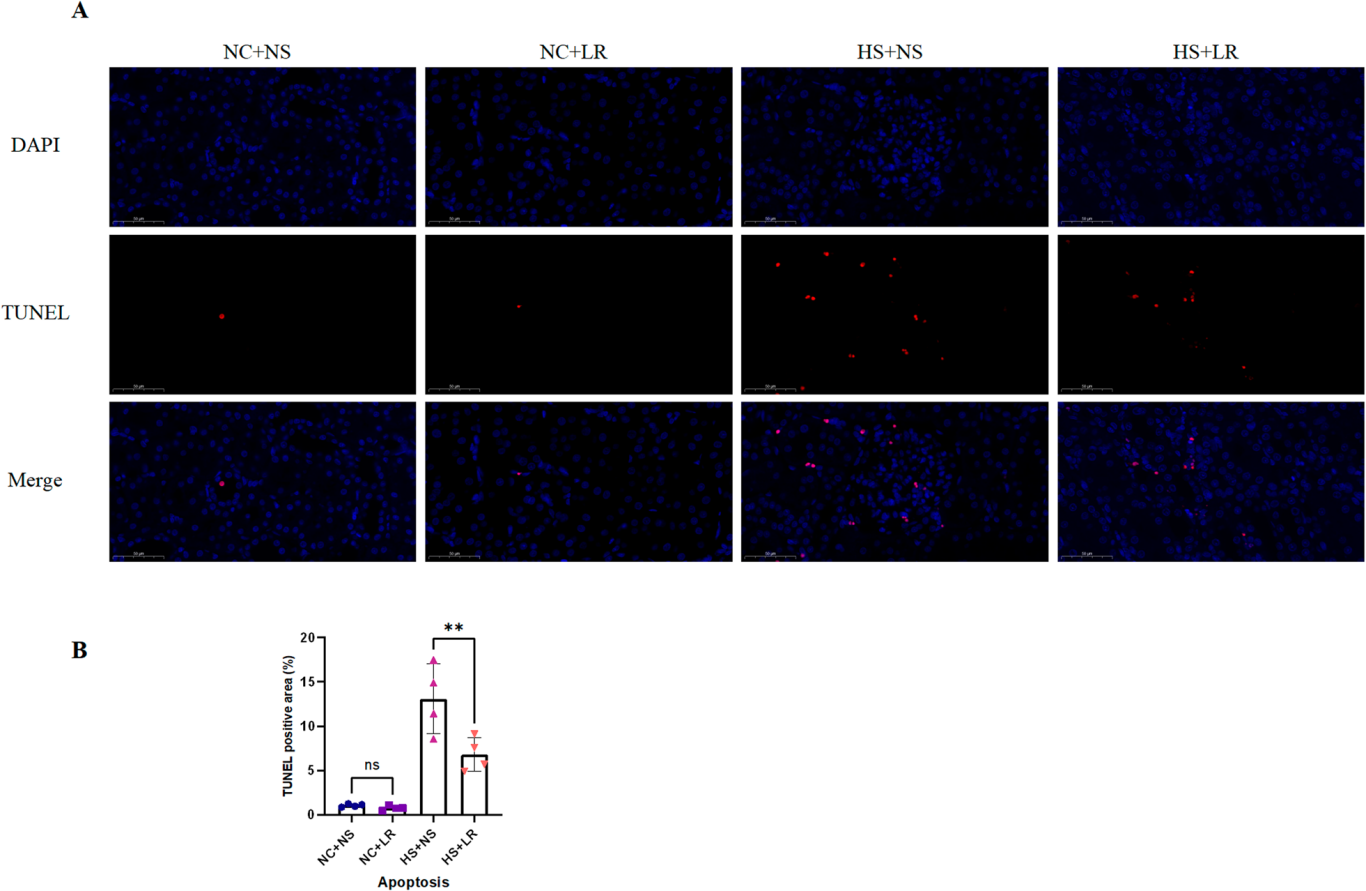
Cell apoptosis of renal cortex tissues evaluated by TUNEL staining. **A** Representative images of TUNEL-stained renal cortex tissue sections. Scale bar = 50 µm. **B** Statistical analysis of the TUNEL-positive area using ImageJ software. Scale bar = 50 µm. All groups *n* = 4. Data are presented as mean ± SD. **P ≤ 0.01

To discern the differential impacts of resuscitation fluids on tissue cell viability, we concurrently assessed apoptosis in kidney tissues across various groups via the TUNEL method. According to the TUNEL assay, the percentage of apoptotic cells was significantly greater in the HS+NS group than in the HS+LR group (12.74 ± 5.18 vs. 4.80 ± 0.83%, *P* = 0.007), as indicated by increased red fluorescence (Fig. 5). These findings suggest that, compared with LRs, NSs exacerbate cellular apoptosis under heat stress.

#### Inflammatory factor expression in serum and tissue

**Fig. 6:**
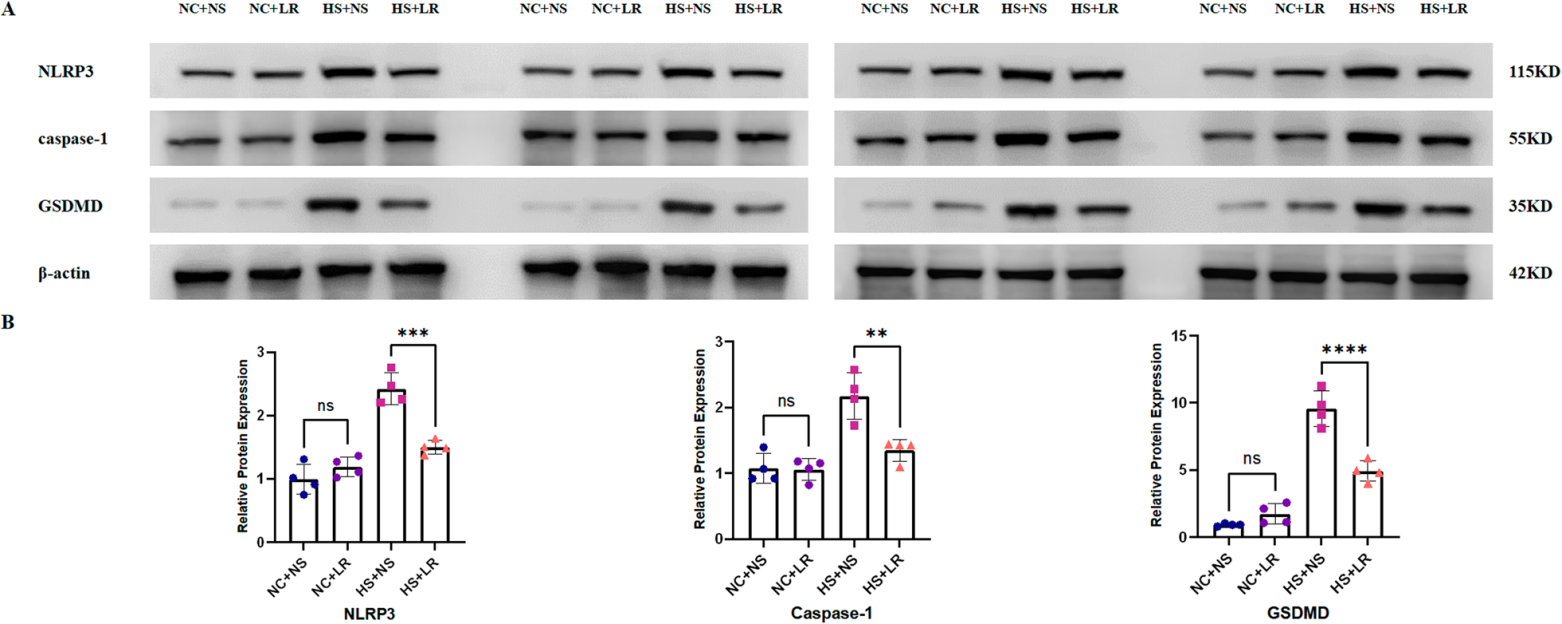
Expression of inflammasome proteins in renal tissues. **A** Protein levels of NLRP3, caspase-1, and GSDMD in kidney tissue, evaluated by Western blotting 2 h after heatstroke onset. **B** Pixel density analysis of the Western blotting results using ImageJ software. Data are presented as mean ± SD. ****P ≤ 0.0001, ***P ≤ 0.001, and **P ≤ 0.01

The WBC count at 2 h posttreatment was significantly greater in the HS+NS group than in the HS+LR group (12.69 ± 2.35 vs. 8.69 ± 2.56, *P* = 0.006). In addition, the HS+NS group presented elevated levels of the inflammatory markers IL-6 and TNF-α in both the serum and cardiac tissues (Table 1), alongside increased expression of the inflammasome proteins NLRP3, caspase-1, and GSDMD in renal tissues (Fig. 6). These observations indicate that NS infusion may exacerbate the inflammatory response in heatstroke, potentially contributing to increased organ dysfunction and mortality rates.

## Discussion

Intravenous fluid infusion critically influences the body's internal environment and is associated with patient prognosis [15]. Despite evidence linking NS to adverse outcomes in critically ill patients, it remains the preferred treatment for heatstroke because of its established clinical use, guideline recommendations, and risk of hyponatremia in heatstroke patients. However, our study revealed that while NS and LR had no significant effects on normal rats, HS+NS rats presented more severe acidosis, increased inflammatory cytokines, and greater renal and cardiac damage than HS+LR rats did. These findings highlight the importance of fluid choice in heatstroke prognosis.

Previous studies have associated high-chloride solutions with metabolic acidosis. NS likely reduces the strong ion difference (SID = Na^+^ + K^+^–Cl^−^), diluting physiological buffers and causing acidosis [26]. Our findings also demonstrated that, compared with the HS+LR group, the HS+NS group presented significantly higher blood chloride levels and a tendency towards metabolic acidosis. This was characterized by low HCO_3_^−^ and PCO_2_, high lactate, and low BE, despite pH compensation remaining within the normal range. Notably, the BE level at 0.5 h postinfusion significantly increased, reaching the diagnostic criteria for severe acidosis (SBE, – 10 to – 15) [14]. In contrast, LR, with chloride concentrations closer to plasma levels and containing lactate, may increase SID and mitigate acidosis. Lactate metabolism to bicarbonate further aids in buffering the pH and stabilizing acid‒base and electrolyte levels [16, 27]. Our results confirm that LR is more effective than NS in maintaining electrolyte and acid‒base balance and that acidosis may trigger inflammatory marker expression and organ damage in HS+NS rats.

High-chloride solutions are also associated with renal-specific vasoconstriction, exacerbated by increased local chloride concentrations [7–9]. Our study revealed slower clearance rates of p-aminohippuric acid and inulin in HS+NS rats than in HS+LR rats, indicating the impact of NSs on renal vascular tension and blood flow. In addition, NS significantly reduced the MAP in normotensive sepsis models with acidosis, likely due to vasodilation and shock mediated by increased nitric oxide (NO) release [22], which aligns with our observations. Although both groups experienced a sharp decrease in MAP after heatstroke, the HS+NS group exhibited a more severe decrease. This hypotension, combined with reduced renal blood flow, exacerbated organ damage, as evidenced by elevated Scr and NGAL levels in HS+NS rats, highlighting the adverse effects of NS on renal function. Abnormal cardiac function indicators may be a consequence of acidosis, hypotension-induced inadequate tissue perfusion, and increased cardiac load due to compensatory mechanisms.

Differential inflammatory responses induced by hydrochloric acid and lactic acid are noteworthy. Hydrochloric acid infusion, which causes moderate-to-severe acidosis (BE < − 5), increases the IL-6-to-IL-10 ratio and TNF synthesis, exerting a proinflammatory effect [14]. Conversely, lactic acid serves as a metabolic regulator and immune signalling molecule, inhibiting lipopolysaccharide-induced cytokine mediators in monocytes and macrophages and negatively regulating the NLRP3 inflammasome and IL-1β production, thus exerting an anti-inflammatory effect [18]. Given the role of inflammation in heatstroke, we explored cytokine expression in rat serum and cardiac tissue. Compared with HS+LR rats, HS+NS rats presented significantly greater levels of inflammatory cytokines (IL-6 and TNF-α), confirming the differential impact of NS and LR on inflammatory responses in heatstroke. The overexpression of inflammatory cytokines exacerbates structural and functional damage to tissues.

Given the aseptic nature of heatstroke-induced inflammation and the critical role of the NLRP3 inflammasome pathway in aseptic inflammation and pyroptotic cell death [18], we examined inflammasome pathway-related proteins in renal tissue via WB. The results revealed that, in NS-treated heatstroke rats, the expression levels of NLRP3, caspase-1, and GSDMD were significantly greater than those in the LR group, further confirming the differential effects of NS and LR on the inflammatory response. NS infusion may promote inflammation by enhancing the activation of the NLRP3/caspase-1/GSDMD pathway, leading to increased cytokine release and subsequent cellular damage.

Histological evaluations confirmed the proinflammatory effects of NSs on organ structure, with the HS+NS group exhibiting more severe inflammatory reactions and cellular damage to heart and kidney tissues. Apoptosis staining revealed numerous TUNEL-positive cells in tubular epithelial regions, glomeruli, and the renal interstitium, suggesting endothelial and inflammatory cell apoptosis (Figure S1), further indicating the proinflammatory effects of NS infusion. To assess the long-term impacts of different fluid regimens, we also conducted histopathological evaluations of renal tissues from heatstroke model rats 24 h postresuscitation. The findings revealed that the severity of damage and necrosis was significantly greater in the HS+NS group than in the HS+LR group (Figure S2). These findings indicate that despite similar Scr levels at 2 h postresuscitation, there were significant differences in structural damage to the kidneys between the two groups, emphasizing the long-term severe impact of NS on renal structure in heatstroke rats.

To our knowledge, this is the first study to directly compare the effects of NS and LR in heatstroke rats. Our findings indicate that NS not only impairs kidney and heart function but also causes significant structural damage to these organs under heatstroke conditions. Furthermore, we identified differential inflammatory regulation by NS and LR, suggesting possible mechanisms involving the inflammasome pathway. Given the critical role of crystalloid infusion in managing heatstroke, our study holds significant value. However, several limitations should be noted. The use of a rat model limits the direct applicability of our findings to human physiology. In addition, our focus was restricted to NS and LR, without exploring the full spectrum of resuscitation fluids. Moreover, despite a thorough investigation of the volume and rate of resuscitation fluids, the infusion rate employed in this study remained substantial relative to the blood volume of the rats. The rapid administration of a large volume of NS may further exacerbate damage in heatstroke-afflicted rats, even though evaluation indicators did not reveal significant differences between the control groups. Our study relied primarily on blood markers and histological evaluations and did not delve deeply into the underlying biochemical mechanisms involved. Future research should aim to validate these findings through human clinical trials and consider a wider range of resuscitation fluids. A deeper understanding of the molecular mechanisms underlying organ dysfunction and inflammatory responses after heatstroke treatment could reveal new therapeutic targets for improving heatstroke management.

## Conclusion

Resuscitation with NS, in comparison to LR, significantly enhances the inflammatory response in heatstroke-afflicted rats, resulting in more severe functional and structural damage to the heart and kidneys. This effect may be mediated by the increased activation of the NLRP3/caspase-1/GSDMD pathway. These findings highlight the critical importance of selecting appropriate resuscitation fluids in heatstroke management to minimize organ damage and improve patient outcomes.

## Supplementary Information

Additional file 1.

## Data Availability

The authors confirm that the data supporting the findings of this study are available at https://doi.org/10.5281/zenodo.11291857.

## Abbreviations

ABG: Arterial blood gases
AKI: Acute kidney injury
ALT: Alanine aminotransferase
BUN: Blood urea nitrogen
CTnI: Cardiac troponin I
ELISA: Enzyme-linked immunosorbent assay
GFR: Glomerular filtration rate
GSDMD: Gasdermin D
HR: Heart rate
H&E: Hematoxylin and eosin
IL-6: Interleukin-6
LR: Lactated Ringer’s solution
MAP: Mean arterial pressure
MODS: Multiple organ dysfunction syndrome
NGAL: Neutrophil gelatinase-associated lipocalin;
NLRP3: NOD-like receptor family pyrin domain containing 3
NS: Normal saline
PLT: Platelet
PT: Prothrombin time
RBF: Renal blood flow
Scr: Serum creatinine
SD: Sprague‒Dawley
Tco: Core body temperature
TEM: Transmission electron microscopy
TNF-α: Tumor necrosis factor-alpha
TUNEL: TdT-mediated dUTP Nick-End Labeling
WBC: White blood cell count
