# Supplementary material for: Effects of normal saline versus lactated Ringer’s solution on organ function and inflammatory responses to heatstroke in rats
Source: J Intensive Care. 2024 Oct 8;12:39. doi: 10.1186/s40560-024-00746-y (PMC11462651; doi:10.1186/s40560-024-00746-y)
Supplement: Supplementary file 1 — Additional file 1. [file 40560_2024_746_MOESM1_ESM.doc]

**Normal Saline versus Lactated Ringer's Solution: Effects on Organ Function and Inflammatory Responses in Heatstroke in Rat**

Lan Chen, Chang Liu, Zhaocai Zhang, Yuping Zhang, Xiuqin Feng

**Table of contents**

[Supplementary Figure 1. 3](#__RefHeading___Toc16974)

[Supplementary Figure 2. 4](#__RefHeading___Toc31563)

[Supplementary Table 1.. 5](#__RefHeading___Toc20581)

**
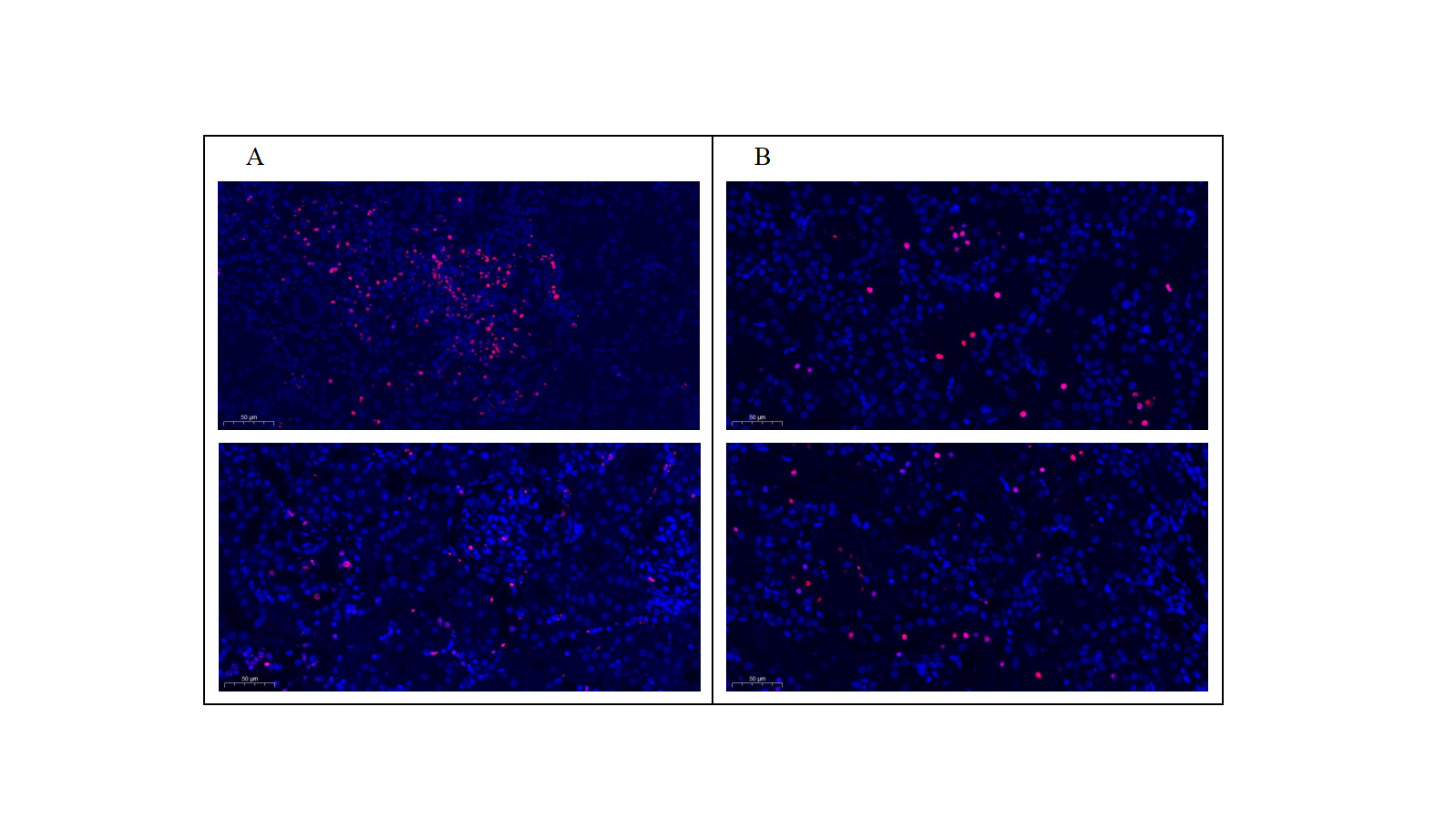
**

**Supplementary Figure 1. Cell Apoptosis of Kidney Tissue Evaluated by TUNEL Staining in the HS+NS Group**

(A) Kidney sections 2 hours after heatstroke onset show TUNEL-positive areas primarily in the glomeruli and renal tubular interstitium, indicating that early cell apoptosis predominantly occurs in endothelial cells or inflammatory cells.

(B) Kidney sections 24 hours after heatstroke onset reveal TUNEL-positive areas in the renal tubular epithelial cells and within the renal tubular lumen, suggesting apoptosis of renal tubular epithelial cells.

**
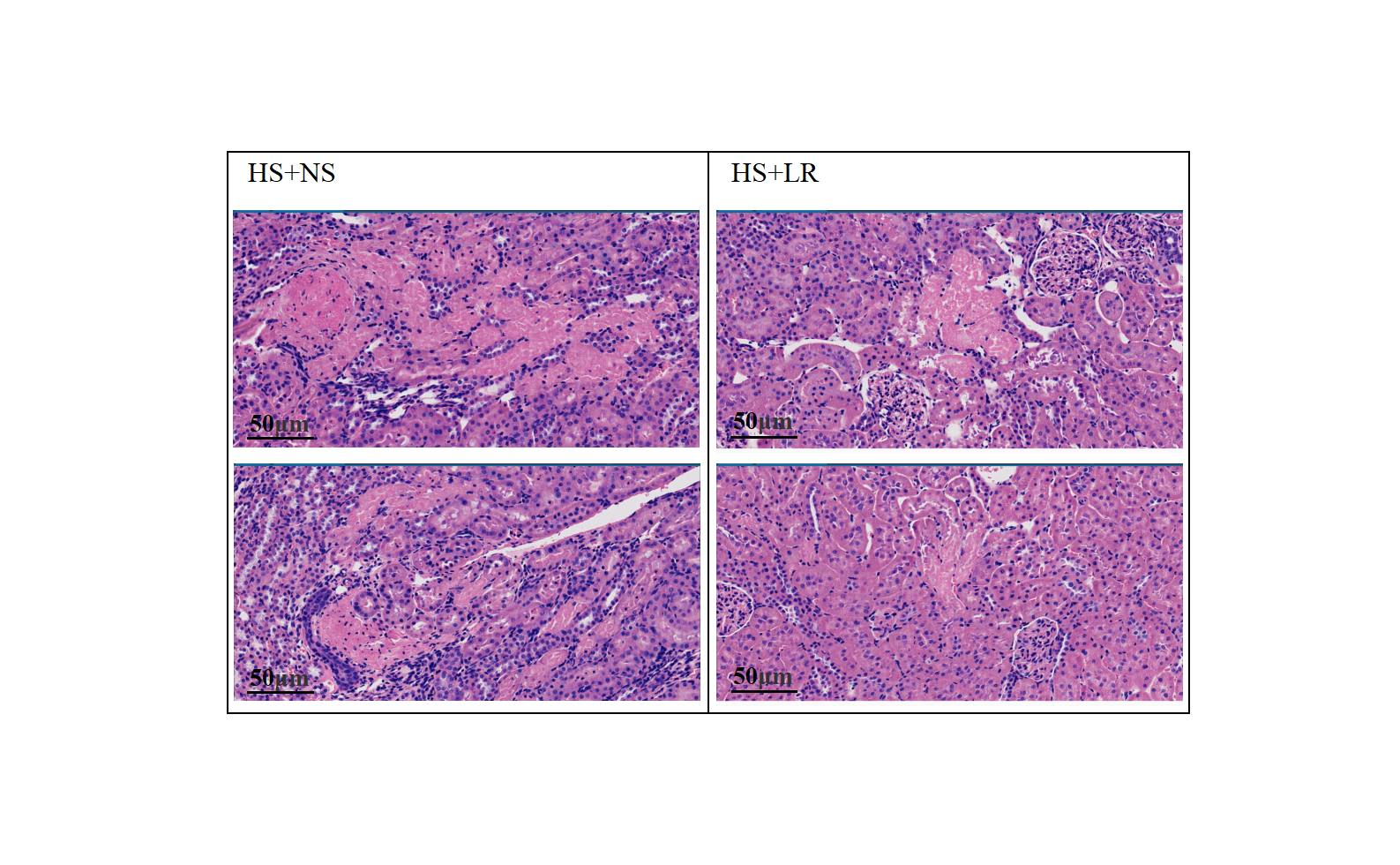
**

**Supplementary Figure 2. Renal Histopathology with H&E Staining at 24 Hours Post-Heatstroke Onset**

In the HS+NS group: Glomeruli and the surrounding proximal tubules displayed coagulative necrosis. Although the outlines of the glomeruli and tubules were still discernible, the epithelial cell boundaries were unclear, characterized by nuclear disappearance, cytoplasmic disintegration, and increased eosinophilia.

In the HS+LR group: The observed renal damage was less severe compared to the HS+NS group.

**Supplementary Table 1. Comparison of Experimental Characteristics Between Four Groups.**

| **Variables** | **NC+NS**  **（n=13)^※^** | **NC+LR**  **（n=14)^※^** | **HS+NS**  **(n=14)^※^** | **HS+LR**  **(n=14)^※^** | ***P-*value^§^** | ***P-*value^＃^** |
| --- | --- | --- | --- | --- | --- | --- |
| **Weight baseline (g)** | 368.35 ± 17.85 | 380.57 ± 15.70 | 369.19 ± 24.19 | 377.47 ± 25.02 | **﹣** | **﹣** |
| **Weight loss (g)** | **﹣** | **﹣** | 3.41 ± 2.51 | 3.60 ± 2.41 | **﹣** | **﹣** |
| **Duration of Tco over 40℃ (min)** | **﹣** | **﹣** | 31.36 ± 5.31 | 32.14 ± 5.14 | **﹣** | **﹣** |
| **Electrolytes and ABG** |  |  |  |  |  |  |
| Sodium (mmol/L) |  |  |  |  |  |  |
| 0.5 h | 139.71 ± 1.25 | 139.43 ± 0.79 | 141.86 ± 1.46 | 140.57 ± 1.51 | **﹣** | **﹣** |
| 2 h | 138.50 ± 2.26 | 139.43 ± 1.99 | 138.86 ± 2.04 | 138.14 ± 1.35 | **﹣** | **﹣** |
| Potassium (mmol/L) |  |  |  |  |  |  |
| 0.5 h | 3.29 ± 0.16 | 3.20 ± 0.17 | 3.74 ± 0.61 | 3.40 ± 0.16 | **﹣** | **﹣** |
| 2 h | 3.32 ± 0.29 | 3.34 ± 0.17 | 3.34 ± 0.26 | 3.41 ± 0.20 | **﹣** | **﹣** |
| PH |  |  |  |  |  |  |
| 0.5 h | 7.36 ± 0.05 | 7.36 ± 0.04 | 7.36 ± 0.05 | 7.40 ± 0.03 | **﹣** | **﹣** |
| 2 h | 7.44 ± 0.04 | 7.41 ± 0.02 | 7.37 ± 0.04 | 7.40 ± 0.06 | **﹣** | **﹣** |
| **Routine blood test** |  |  |  |  |  |  |
| PLT (10^9/L) |  |  |  |  |  |  |
| 0.5 h | 541.14 ± 135.40 | 529.29 ± 74.39 | 574.86 ± 62.08 | 537.71 ± 108.59 | **﹣** | **﹣** |
| 2 h | 500.67 ± 61.11 | 574.00 ± 44.71 | 558.71 ± 84.27 | 539.57 ± 63.94 | **﹣** | **﹣** |
| **coagulation function** |  |  |  |  |  |  |
| PT (S) |  |  |  |  |  |  |
| 0.5 h | 10.69 ± 1.06 | 10.99 ± 0.49 | 11.99 ± 0.85 | 11.31 ± 0.71 | **﹣** | **﹣** |
| 2 h | 11.40 ± 0.73 | 11.01 ± 0.77 | 11.71 ± 0.45 | 10.76 ± 1.81 | **﹣** | **﹣** |
| **Liver function** |  |  |  |  |  |  |
| ALT (U/L) |  |  |  |  |  |  |
| 0.5 h | 30.23 ± 5.77 | 27.94 ± 9.25 | 32.54 ± 4.40 | 35.31 ± 4.88 | **﹣** | **﹣** |
| 2 h | 34.47 ± 5.42 | 32.23 ± 4.26 | 51.21 ± 8.92 | 48.24 ± 2.46 | **﹣** | **﹣** |
| **Renal function** |  |  |  |  |  |  |
| BUN (mmol/L) |  |  |  |  |  |  |
| 0.5 h | 4.39 ± 0.93 | 4.10 ± 0.42 | 7.37 ± 0.91 | 6.81 ± 0.69 | **﹣** | **﹣** |
| 2 h | 3.87 ± 0.25 | 4.30 ± 0.62 | 8.12 ± 1.43 | 7.98 ± 0.71 | **﹣** | **﹣** |

All specimens are blood samples.

Data are represented as the mean ± SD.

Group Sample Sizes: NC+NS: n=13 (7 in 0.5 h, 6 in 2 h); NC+LR, HS+NS, and HS+LR: n=14 (7 in 0.5 h, 7 in 2 h);.

Comparisons: P^§^: NC+NS vs. NC+LR; P^＃^: HS+NS vs. HS+LR.

ABG: arterial blood gases; BUN: blood urea nitrogen; PLT, platelet; PT, prothrombin time.
